# Hydatidose du muscle psoas révélée par une compression de l'axe vasculaire du membre inférieur : À propos d'un cas au Centre hospitalier universitaire Ibn Sina, Rabat, Maroc

**DOI:** 10.48327/mtsi.v2i3.2022.195

**Published:** 2022-07-18

**Authors:** Manal BOUIKHIF, Sana FARHANE, Mohamed LYAGOUBI, Sarra AOUFI

**Affiliations:** 1 Laboratoire central de parasitologie et mycologie, Centre hospitalier universitaire Ibn Sina, Rabat, Maroc; 2 Faculté de médecine et de pharmacie, Université Mohamed V, Rabat, Maroc

**Keywords:** Pays endémique, Hydatidose, Muscle psoas, Insuffisance veineuse, *Echinococcus granulosus*, Maroc, Maghreb, Afrique du Nord, Endemic countries, Hydatidosis, Psoas muscle, Venous insufficiency, *Echinococcus granulosus*, Morocco, Maghreb, Northern Africa

## Abstract

**Introduction:**

L'hydatidose est une parasitose endémique au Maroc. Elle touche le foie, les poumons et rarement les muscles. L'hydatidose du psoas reste inhabituelle, même dans les pays endémiques.

**Observation:**

Un patient s'est présenté en consultation de chirurgie vasculaire pour une douleur de l'hypochondre gauche avec une distension abdominale et une lourdeur du membre inférieur gauche. Une tomodensitométrie injectée du pelvis avait montré une masse kystique du muscle psoas gauche dont la description radiologique évoquait une hydatidose qui comprimait l'axe vasculaire iliaque. Cette suspicion a été confirmée par une sérologie hydatique positive par deux techniques différentes. Le patient a été opéré pour exérèse du kyste et son contenu envoyé au laboratoire de parasitologie qui a objectivé la présence d’*Echinococcus granulosus*. Un an après, le patient a présenté une récidive de son kyste.

**Conclusion:**

La localisation isolée du kyste hydatique au muscle posas est rarissime. Son diagnostic repose sur les données épidémiologiques, cliniques, radiologiques et est confirmé par la sérologie et l'examen parasitologique de la pièce opératoire.

## Abstract (en)

**Introduction:**

Hydatidosis is a zoonosis caused by *Echinococcus granulosus*. It's a real public health problem in Morocco. Muscle localization is unusual, even in endemic countries. We report a rarely described case of a hydatid cyst of the psoas muscle diagnosed in our laboratory.

**Case report:**

The patient was a 32-year-old male, living in a rural area. He reported a contact with dogs. He was admitted in vascular surgery department for left hypochondrium pain, with heaviness of the left lower limb and appearance of varicose veins. Clinical examination has found a huge painful and hard mass of the left flank arriving to the hypogastrium with varicose veins of the left leg. An injected CT scan of the pelvic region showed a 189 x 137 mm cystic mass of the left psoas muscle reflowing left iliac vessels. This suggested hydatid cyst. No other localization was found. Hydatid serology was positive with an ELISA test and an indirect hemagglutination test. The patient underwent surgery to remove the mass. Several white vesicles of a few centimeters were found in the cyst and were sent to the parasitology laboratory. Microscopic examination has confirmed the presence of viable *Echinococcus granulosus*. The patient received albendazole 400 mg twice daily for only a month and was not seen for follow-up. One year after surgery, he showed the same symptoms of abdominal pain and heaviness. Recurrence of hydatid cysts in the same localization was diagnosed with ultrasound showing two hydatid cysts type 3 according to Gharbi classification.

**Discussion:**

Diagnosis of all hydatidosis localizations is based on epidemiological, clinical, and radiological data and confirmed by serology and parasitological examination of the surgical specimen. Surgery is then a diagnostic and therapeutic tool that cannot be bypassed since it allows the definitive elimination of the parasite and eviction of recurrence if it's well done. Conservative methods are related to recurrent cysts.

**Conclusion:**

Muscle hydatidosis is extremely rare but should not be forgotten when radiological and epidemiological data suggest it. It's a benign infection but can be severe and deadly if not appropriately treated.

## Introduction

L'hydatidose est une parasitose due au développement chez l'homme du stade larvaire d’*Echinococcus granulosus*, cestode de petite taille qui vit dans l'intestin grêle des canidés. Cette parasitose constitue un véritable problème de santé publique dans les pays d’élevage pastoral du bassin méditerranéen et surtout au Maroc. Le poumon est sa localisation préférentielle après le foie, et les muscles sont rarement touchés [5]. Nous rapportons un cas d'hydatidose du muscle psoas, localisation inhabituelle, révélée par une compression vasculaire unilatérale du membre inférieur.

## Description Clinique et Paraclinique

**Figure 1:**
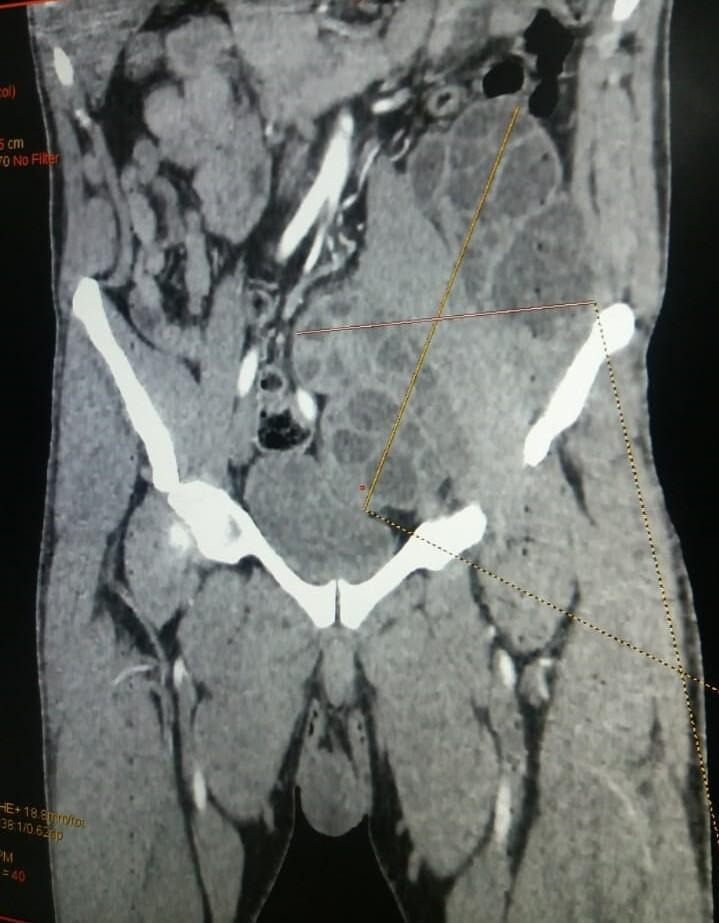
Coupe sagittale de tomodensitométrie injectée abdominopelvienne montrant une énorme masse kystique multicloisonnée évoquant une hydatidose Sagittal section of an injected abdominal-pelvic CT scan showing a huge multiclonal cystic mass suggestive of hydatidosis

Un patient de sexe masculin âgé de 32 ans vivant en milieu rural dans la région de Khouribga située au sud-est de Casablanca a consulté au service de chirurgie vasculaire. Il se plaignait d'une douleur chronique depuis un an du flanc gauche à type de pesanteur, sans irradiation, avec une distension abdominale progressive qui s'est compliquée d'une lourdeur du membre inférieur gauche et de gonflement du membre après l'effort. Le patient a décrit la présence de varicosités sur la face interne de la jambe. À l'interrogatoire, le patient a rapporté un contact avec des chiens. L'examen clinique a objectivé une énorme masse douloureuse et dure du flanc gauche arrivant à l'hypogastre avec un oedème de la cuisse gauche et des varices alors que tous les pouls étaient présents. Devant ce syndrome de masse pelvienne avec des signes de compression vasculaire unilatérale, une angio-tomodensitométrie a été réalisée et a montré une énorme masse abdomino-pelvienne, de tonalité hydrique multiloculaire, se développant aux dépens du muscle psoas iliaque gauche étendue en rétropéritonéal et lysant la crête de l'os iliaque en regard. Elle mesurait 189 mm sur 137 mm (Fig. 1 et 2), exerçait un effet de masse sur la vessie et refoulait l'axe vasculaire iliaque gauche. L'image radiologique évoquait une hydatidose. Aucune autre localisation, notamment hépatique, n'a été retrouvée. Une sérologie hydatique a été demandée au laboratoire de parasitologie. Un test à ELISA (Echinococcus IgG ELISA, DRG^®^) est revenu positif à 52,33 UI/ml (seuil de positivité de 11 UI/ml) de même que l'hémagglutination indirecte (Hydatidose Fumouze, Biosynex^®^) qui était supérieure à 1/640. Le patient a été ainsi proposé pour traitement chirurgical. Une périkystectomie totale par voie extrapéritonéale par une incision type Jalaguier gauche a été réalisée en utilisant des champs opératoires imbibés d'eau oxygénée, précédée par une prise de 400 mg d'albendazole par voie orale. Cette voie d'abord chirurgicale a été privilégiée pour éviter l'ouverture du péritoine qui exposerait au risque d'hydatidose péritonéale en cas de rupture du kyste. En per-opératoire, on découvre une unique formation kystique énorme en contact étroit avec le psoas et l'axe vasculaire iliaque gauche. L'ouverture accidentelle de la masse kystique a fait découvrir plusieurs formations arrondies blanches nacrées qui ont été extraites en totalité et adressées au laboratoire de parasitologie pour identification. L'examen macroscopique de la pièce reçue a trouvé plusieurs vésicules à paroi blanche mesurant chacune entre 2 et 5 cm de diamètre. Ouvertes, elles contenaient un liquide eau de roche. Le liquide a été centrifugé après un raclage de la face interne des vésicules par une lame bistouri. L'examen microscopique du culot a révélé la présence de plusieurs protoscolex invaginés et dévaginés portant chacun une double couronne de crochets (Fig. 3). Leur viabilité a été démontrée par leur mobilité et un test à l’éosine 0,2% qui a montré plusieurs protoscolex résistants à la coloration (Fig. 4), confirmant le diagnostic d’*Echinococcus granulosus*. Le traitement par albendazole 400 mg en deux prises par jour a été maintenu en post-opératoire mais a été arrêté après un mois par le patient qui a ensuite été perdu de vue.

**Figure 2:**
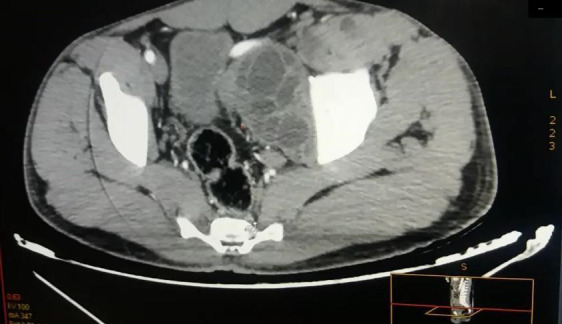
Coupe transversale de tomodensitométrie injectée abdomino-pelvienne montrant une énorme masse au dépend du muscle psoas refoulant la vessie Cross-section of injected abdominal-pelvic CT scan showing a huge mass at the psoas muscle pushing back the bladder

**Figure 3:**
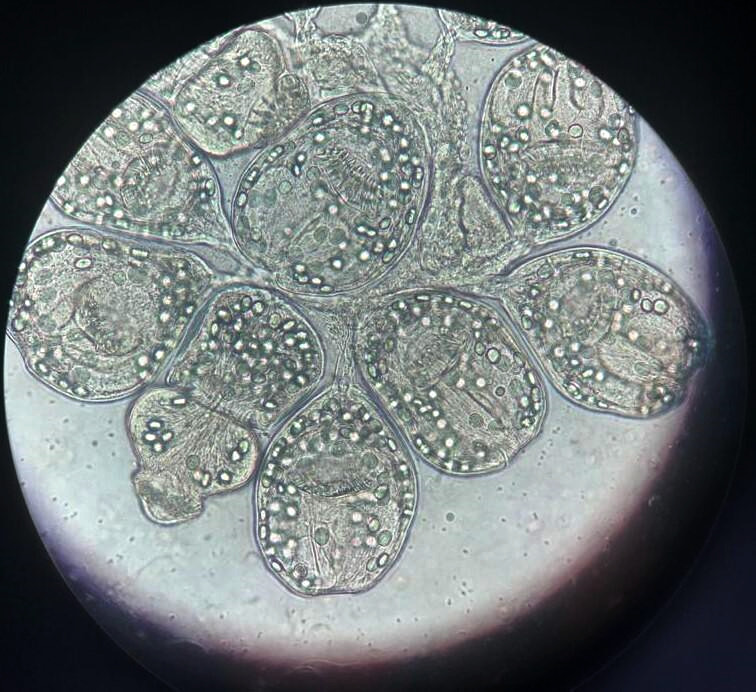
Vue microscopique à l'objectif 10x du culot de centrifugation montrant de nombreux protoscolex invaginés et dévaginés portant chacun une double couronne de crochets confirmant la présence d’*Echinococcus granulosus* *Microscopic view at 10x objective of the centrifugation pellet showing numerous invaginated and evaginated protoscoleces with a double crown of hooks on each confirming* Echinococcus granulosus

**Figure 4:**
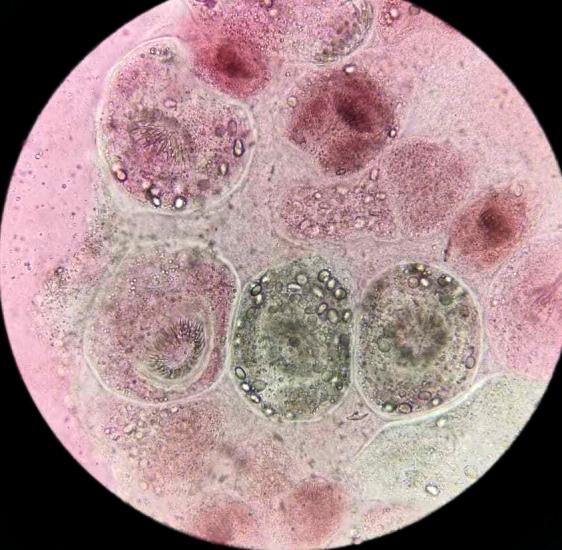
Vue microscopique d'une préparation du culot de centrifugation par l’éosine à 2% montrant des protoscolex d’*Echinococcus granulosus* viables réfractaires à la coloration *Microscopic view of eosin-exclusion test showing viable protoscoleces of* Echinococcus granulosus

Un an plus tard, le patient a consulté pour la réapparition des symptômes initiaux faits de douleur et pesanteur abdominales. Une échographie a été réalisée et a montré une récidive de la parasitose en deux kystes hydatiques sur le muscle psoas, classés type 3 selon Gharbi *et al.* [10]. Le patient a été remis sous albendazole en vue d'une reprise chirurgicale de la récidive.

## Discussion

L'hydatidose ou échinococcose est une parasitose cosmopolite mais qui sévit à l’état endémique dans les pays où l’élevage et l'abattage traditionnels sont fréquents [5]. L'homme ne constitue qu'un hôte intermédiaire accidentel et une impasse parasitaire puisqu'il ne transmet pas la maladie [9]. La physiopathologie des localisations musculaires reste encore mal élucidée. Le muscle ne constitue pas un lieu favorable pour l'accueil et le développement de la larve hydatique par sa contraction et la production d'acide lactique [13]. Une localisation au niveau du muscle psoas a été rapportée dans plusieurs cas dont la plupart étaient primitifs du muscle [1, 4, 7, 8, 11]. Les localisations du psoas sont révélées par l'augmentation du volume de l'hydatide, par une douleur [11] ou masse palpable [4], ou par la compression nerveuse ou la compression vasculaire comme chez notre patient. L'imagerie permet la reconnaissance du kyste hydatique [14], comme pour notre patient, sans avoir recours à des manoeuvres invasives qui pourraient disséminer les larves avec le risque de créer autant de nouveaux kystes. La sérologie a une valeur prédictive importante mais malheureusement est souvent négative [13, 15]. Elle permet essentiellement de confirmer la nature hydatique d'une image radiologique suspecte, et présente une sensibilité et une spécificité accrues quand il s'agit de localisations hépatiques, mais moindres pour les poumons et les autres localisations [15]. Cependant, une série de 9 cas de kystes hydatiques du psoas rapportée par des auteurs tunisiens a montré que la sérologie hydatique a été positive chez 3 patients sur 4 pour qui elle était demandée [4]. Toutefois, la chirurgie est un moyen diagnostic et aussi thérapeutique qui conduit à l’élimination du parasite en évitant sa dissémination. L'albendazole est l'antihelminthique de référence pour le traitement médical de l'hydatidose réservé aux patients inopérables ou aux formes inextirpables [3]. L'administration de l'albendazole en pré-opératoire est nécessaire pour éviter une dissémination en cas d'incident peropératoire. La dose chez l'adulte est de 15 mg/kg/j sans dépasser 800 mg/j en deux prises. La durée de traitement pré-opératoire est variable entre 1 [17] et 6 mois [6] selon les études. Cette chiomioprophylaxie consiste essentiellement à réduire la pression intrakystique pour faciliter son exérèse chirurgicale et à restreindre significativement la viabilité des scolex et donc le risque de récidive post-opératoire de l'hydatidose [2]. Malheureusement, ces recommandations n'ont pas été respectées chez notre patient, ce qui a été confirmé immédiatement en pré-opératoire par la mise en évidence des protoscolex vivants au test à l’éosine et ultérieurement par la récidive in situ du parasite. Les récidives de kystes hydatiques sont connues et décrites dans la littérature. Elles constituent la complication post-opératoire tardive la plus fréquente avec des taux variables selon les séries [12, 16, 18]. Ces récidives sont principalement conditionnées par le déroulement des méthodes thérapeutiques, les techniques conservatrices comme la ponction-aspiration du kyste hydatique et la résection partielle étant les plus liées à un taux de récidive élevé ainsi que l'ouverture accidentelle du kyste hydatique en per-opératoire comme pour notre patient [16]. Ce risque de récidive justifie une surveillance étroite à la fois clinique, sérologique et radiologique pour une longue période. Cette surveillance a été défaillante chez notre patient puisqu'il était perdu de vue après l'opération et n'a reconsulté que tardivement après l'apparition de la récidive.

## Conclusion

L'hydatidose est une parasitose qui constitue encore un problème de santé dans les pays moins développés. Sa prévention fait l'objet de plusieurs programmes de lutte nationaux au Maroc et internationaux sous l’égide de l'OMS. Le diagnostic pré-opératoire d'un kyste hydatique et le choix des méthodes thérapeutiques conditionnent le pronostic des patients puisque les récidives et disséminations sont associées à une augmentation de la morbi-mortalité.

## Liens D'intérêts

Les auteurs ne déclarent aucun lien d'intérêt.

## Contribution des Auteurs

Dr Manal BOUIKHIF : conception, recueil des données, rédaction, correspondance. Dr Sana FARHANE : recueil des données. Pr Sarra AOUFI : rédaction et correction du manuscrit.

Pr Mohamed LYAGOUBI : relecture et validation du manuscrit.
